# Assessment of Oral Mucosal Colour in Horses: Initial Application of the Uldahl Standard Across Physiological States

**DOI:** 10.3390/ani16213471

**Published:** 2026-11-03

**Authors:** Mette Uldahl, Jan Dahl, David J. Mellor

**Affiliations:** 1 BrightHorses Institute, Fasanvej 12, 7120 Vejle Oest, Denmark; 2 Independent Researcher, Oestrupvej 89, 4350 Ugerloese, Denmark; jd@lf.dk; 3 Animal Welfare Science and Bioethics Centre, School of Veterinary Science, Massey University, Palmerston North 4442, New Zealand; d.j.mellor@massey.ac.nz

**Keywords:** horse, equine, oral mucosae, mucosal colour, physiological state, sedation, circulatory compromise, welfare assessment, veterinary diagnostics

## Abstract

Assessment of oral mucosal colour is widely used in equine clinical and welfare evaluations, but inconsistent terminology limits comparison between clinical observations and research studies. This study evaluates, as an initial proof-of-concept, the Uldahl Standard for systematically describing mucosal colour and saturation. Gingival and lingual mucosae were assessed in clinically normal horses, healthy horses sedated for routine dental procedures, and horses with severe systemic circulatory compromise. Photographic colour samples validated against in vivo observations were classified using Munsell notation, ISCC-NBS terminology and the Uldahl Standard. Three illustrative cases of tongue discolouration were additionally assessed. Descriptive analyses characterised colour and saturation across physiological states, while clinically normal and sedated horses were compared using exact chi-square tests and logistic regression. Overall, 42 unique colour-saturation combinations were identified. Chromatic diversity increased with physiological compromise. Primary colour differed significantly between clinically normal and sedated horses for both gingival (*p* = 0.05) and lingual (*p* = 0.004) mucosae, whereas secondary colour and saturation did not. Grey was significantly associated with physiological state in both gingival (*p* = 0.05) and lingual (*p* = 0.03) mucosae. It showed high specificity but low sensitivity, including 100% specificity for lingual grey. Purple occurred only in sedated and systemically compromised horses, while blue occurred only as a secondary colour in systemically compromised horses. The findings demonstrate the feasibility of applying the Uldahl Standard to equine oral mucosal assessment and suggest that primary colour, particularly grey, may provide greater discriminatory information than secondary colour or saturation. Further studies are required to establish the diagnostic relevance of broader colour-saturation patterns.

## Simple Summary

Oral mucosal colour is routinely assessed in horses because changes in colour may provide information about circulation, oxygenation, health and welfare. However, descriptions of mucosal colour are inconsistent, making comparisons between clinical observations and scientific studies difficult. This study provides an initial evaluation of the Uldahl Standard, a framework developed to standardise descriptions of mucosal colour and saturation. Gingival and lingual mucosal colours were assessed in clinically normal, non-sedated horses, sedated horses and horses with severe systemic circulatory compromise, together with three illustrative cases. Overall, 42 different colour-saturation combinations were identified. Colour variation increased with physiological compromise, and primary colour more effectively distinguished clinically normal from sedated horses than did secondary colour or saturation. Grey showed particularly high specificity for physiological alteration, while purple and blue were observed only in physiologically compromised horses within the dataset. The findings demonstrate that the Uldahl Standard can describe equine mucosal colour systematically and identify colour characteristics that may warrant further investigation as indicators of physiological change. However, studies of more precisely described physiological and pathophysiological changes are needed to determine the diagnostic significance of specific colour patterns to further improve their potential application in clinical and welfare assessment.

## 1. Introduction

Assessment of oral mucosal colour is a routine component of veterinary examination [1,2], and provides information regarding circulatory function, oxygenation, health status, and welfare in terrestrial mammals, including horses [1,3,4,5,6]. Gingival and lingual colours are also incorporated into welfare assessment systems, including the Standardised Equine-Based Welfare Assessment Tool (SEBWAT) [7,8]. As mucosal colour may reflect both local and systemic physiological or pathological changes, it can contribute to clinical decision making [1,9,10,11,12,13,14,15].

Normal and abnormal equine mucosal colours are widely described in textbooks, clinical manuals, welfare assessment protocols and scientific publications [1,6,8,9,10,11,12,13,14,15,16,17,18,19,20,21,22,23,24,25,26]. Pink is generally associated with adequate perfusion and oxygenation [1,17,18,20,23,24,27], whereas red, blue, purple, yellow, brown and grey have been associated with conditions including inflammation, circulatory compromise, hypoxia, shock, endotoxemia, hepatic disease and tissue degeneration [1,3,6,9,10,11,13,14,15,17,18,19,20,22,24,25,26,27,28,29]. Patchy and pharmacologically induced colour changes have also been reported [6,29].

However, colour terminology is inconsistent. Formal descriptors such as pale, vivid, deep and dark coexist with less precisely defined terms including brilliant brick, injected, muddy, toxic, congested and diffuse [1,6,9,10,11,13,17,18,19,20,23,24,27,28]. Even commonly used descriptors are interpreted differently; pale pink is regarded as normal in some sources but abnormal in others [1,10,17,18,20,23,24,27]. Surface characteristics such as moist, shiny, dry, sticky and clammy are additionally used alongside colour descriptors [1,10,19]. Such variation limits comparison between studies and complicates clinical and scientific communication.

The Uldahl Standard was developed to address these limitations by defining distinct colour categories and saturation levels for mucosal colour assessment in terrestrial mammals [5]. All major colour categories described in the equine literature are represented within the framework. Standardised assessment may be particularly relevant where changes in mucosal colour reflect physiological or pathological disturbances and potentially compromised welfare.

One such context is ridden horses, where gingival and lingual colour changes have been observed during exercise and in association with bit use, including tongues described as blue, dark blue, or black [24,30]. Proposed mechanisms include local circulatory restriction from bit pressure [31] and reduced respiratory efficiency associated with head and neck positions [32,33,34,35]. The tongue becomes visible when the mouth opens, a behaviour associated with bit-related pain and lesions [30,36,37,38]. In obligate nasal breathers, mouth opening may also further compromise airflow [20,23,32,35,39]. Combined with the tongue’s high sensitivity and nociceptive capacity [23,24,30,39], such observations have important welfare implications. These observations emphasise the need to understand normal mucosal colour variation and deviations from it.

Physiological variation is also relevant when mucosal colour is used as a research outcome. Acute intense exercise has been associated with transient changes in mucosal appearance in otherwise healthy horses [40], while altered appearance has been reported with poor recovery or metabolic compromise following prolonged exercise [41,42]. Although age influences cardiovascular and thermoregulatory physiology [43], there is insufficient evidence that age per se systematically alters perfusion-related oral mucosal colour, and evidence of systematic effects of other physiological variables, including hormonal cycles, is similarly limited. Such studies, incorporating mucosal colour, while examining parameters like the effects of exercise and age, have used heterogeneous terminology and assessment methods, frequently without standardised colour definitions [41,42,43]. Collectively, the available evidence supports an association between physiological state and mucosal appearance, while the need for standardised assessment and reporting is evident.

Sedation provides a potentially useful model for investigating medically induced changes. Commonly used sedative protocols produce measurable physiological changes, including reductions in heart rate, cardiac output and oxygen delivery; changes in systemic vascular resistance and arterial blood pressure; and alterations in pulmonary gas exchange and arterial oxygenation. These effects have been demonstrated in horses injected with detomidine and butorphanol [44,45,46]. Although ⍺-2 agonist-mediated vasoconstriction has been described as potentially producing a pale or greyish appearance [25], corresponding changes in equine oral mucosal colour have not been systematically characterised.

The present study represents an initial proof of concept rather than an exhaustive diagnostic classification. It applies the Uldahl Standard to equine oral mucosal colour data and illustrative clinical cases to determine whether the framework can systematically characterise colour and saturation and identify potentially informative colour characteristics. Clinically normal horses represented the physiologically unaffected reference population, while sedated healthy horses represented a controlled pharmacologically altered physiological state with documented cardiovascular and respiratory effects [44,45,46]. For the descriptive part of the study, horses with severe systemic circulatory compromises associated with underlying diseases represented pathological states. Selected cases of tongue discolouration were additionally included to demonstrate Uldahl Standard applications under specified conditions.

A veterinarian-led clinical visual assessment approach was applied in the study, supplemented by generative AI for more detailed colour characterisation within the Uldahl Standard, consistent with emerging human-AI collaborative approaches in which generative AI supports rather than replaces expert assessment [5,47,48,49,50]. In this proof-of-concept study, photographs were not treated as independent objective measurements of mucosal colour; rather, direct veterinary assessment served as the clinical reference for validating the photographic representation, consistent with approaches used in other clinical fields where expert in-person assessment has been used as the reference against which photographic representations are evaluated [51,52,53,54,55].

The aims were to: (1) describe gingival and lingual colour and saturation characteristics across the three physiological groups; and (2) to determine in clinically normal horses and sedated horses identifiable colour or saturation characteristics associated with altered physiological states.

## 2. Materials and Methods

### 2.1. Key Features of the Uldahl Standard

The Uldahl Standard applies defined colourimetric principles and the ISCC-NBS colour naming system to mucosal colour assessment [5]. Mucosal appearance is characterised by three integrated parameters: (1) colour, assigned to nine categories (yellow, green, blue, purple, pink, red, orange, brown, grey), with optional secondary hues for composite colours; (2) saturation, classified as pale, light, moderate, strong, vivid, deep, dark and very dark; and (3) physiological association, linking observed colour characteristics to physiological context.

Additional assessment parameters include species, anatomical site, health status, environmental conditions and artefacts.

Assessment may be visual, combined visual and computational, or fully computational or instrumental, with the level of validation reported according to the method used [5]. These approaches represent different levels of assessment rather than interchangeable measures. In a clinical visual approach, direct in vivo assessment by the veterinarian may serve as the reference for validating whether a photographic representation adequately reflects the observed mucosal colour, whereas studies designed for objective or quantitative colour measurement may additionally incorporate standardised illumination, colour calibration and instrumental measurements.

### 2.2. Application of a Conventional Clinical Visual Approach Supported by Generative AI

The study design was based on a conventional clinical visual approach to mucosal colour assessment. Veterinarians first assessed the oral mucosal colour in vivo and then compared this with photographs obtained immediately after the clinical assessment to confirm that the photographic representation adequately reflected the colours observed in vivo. Only photographs considered representative of the corresponding in vivo appearance were included for subsequent analysis.

At the analytical stage, visual assessment and allocation of gingival and lingual photographic samples to the principal colour and saturation categories were performed by a veterinarian (MU). Generative AI was used as a secondary supportive tool to facilitate more detailed interpretation of colour characteristics and their relation to Munsell notation and ISCC-NBS colour terminology according to the Uldahl Standard.

### 2.3. Study Design

The study comprised three groups of horses representing different physiological states and three illustrative cases. It was conducted in six sequential phases: (1) Collection of veterinary-validated photographs of gingival and lingual mucosae from clinically normal, sedated horses, and horses with systemic pathology; (2) extraction and classification of colour samples according to Munsell notation, ISCC-NBS terminology and the Uldahl Standard; (3) quality control and repeated assessment of borderline and inconsistent classifications; (4) descriptive analyses of colour and saturation within the three groups; (5) statistical comparative and logistic regression analyses of clinically normal and sedated horses; and (6) evaluation of three illustrative cases.

### 2.4. Study Populations and Data Collection

Eleven equine veterinarians recruited through dedicated professional social media networks collected data according to written instructions. Horses constituted a convenience sample of animals encountered by the participating veterinarians during routine clinical work. For inclusion in either of the two healthy groups (sedated and non-sedated), the principal criterion was the attending veterinarian’s clinical assessment that the horse was healthy at the time of examination; no further subgroup criteria were applied. Between September and November 2024, 121 horses were evaluated: 63 clinically normal non-sedated horses, 53 clinically healthy horses sedated for routine dental procedures, and five horses with systemic pathology.

**Figure 1:**
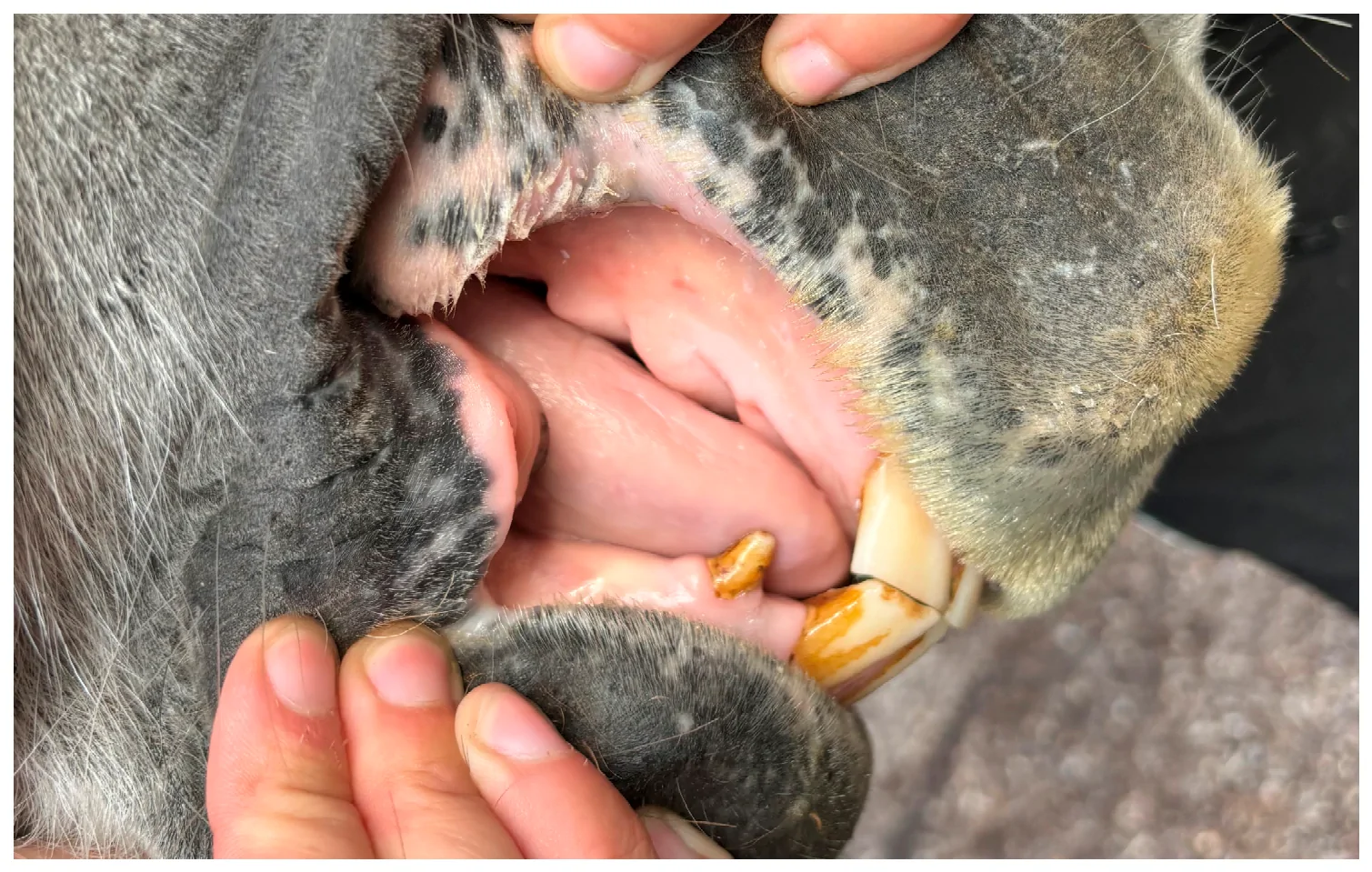
Separating the upper and lower lips at the lateral aspect of the mouth to allow visualisation of both the gingival and lingual mucosa.

For all study groups, veterinarians separated the upper and lower lips laterally to visualise the gingival and lingual mucosae (Figure 1), assessed mucosal colour directly in vivo, and photographed the same anatomical region.

Photographs were obtained using the participating veterinarians’ smartphones, with the camera positioned approximately 15–30 cm from the horse’s mouth. Following image acquisition, the veterinarian compared the photograph with the mucosal colour observed directly in vivo. Photographs were included only when the attending veterinarian considered that the mucosas were adequately visualised for clinical assessment and confirmed that the mucosal colours represented photographically corresponded to those observed in vivo. Images that did not meet these criteria were not included in the study.

Horse identity and confirmation of colour agreement accompanied each submitted photograph, along with evidence that prior owner consent was obtained.

In sedated horses, assessment and photography were performed 5–30 min after intravenous administration of approximately 0.025 mg/kg butorphanol (10 mg/mL) and 0.012 mg/kg detomidine during established sedation. This combination has been associated with reductions in heart rate, cardiac output and oxygen delivery, alterations in vascular resistance and arterial blood pressure, and impaired pulmonary gas exchange and arterial oxygenation [44,45,46].

The five horses with systemic pathology were examined during clinical presentation with shock associated with underlying disease; two had strangulating intestinal obstruction, one had an intestinal rupture, one had mesenteric infarction, and one had abscess formation. These conditions represented severe systemic circulatory compromises associated with gastrointestinal disease, intestinal ischaemia, systemic inflammatory response and endotoxaemia [56,57,58,59,60]. The inclusion criteria for this group were a veterinary examination diagnosing an acute severe pathological condition affecting systemic circulation and requiring immediate veterinary intervention or euthanasia. All horses were assessed under resting conditions and had not been exercised immediately prior to data collection.

All horses were identified by the veterinarians using assigned unique identity numbers.

### 2.5. Individual Cases

**Figure 2:**
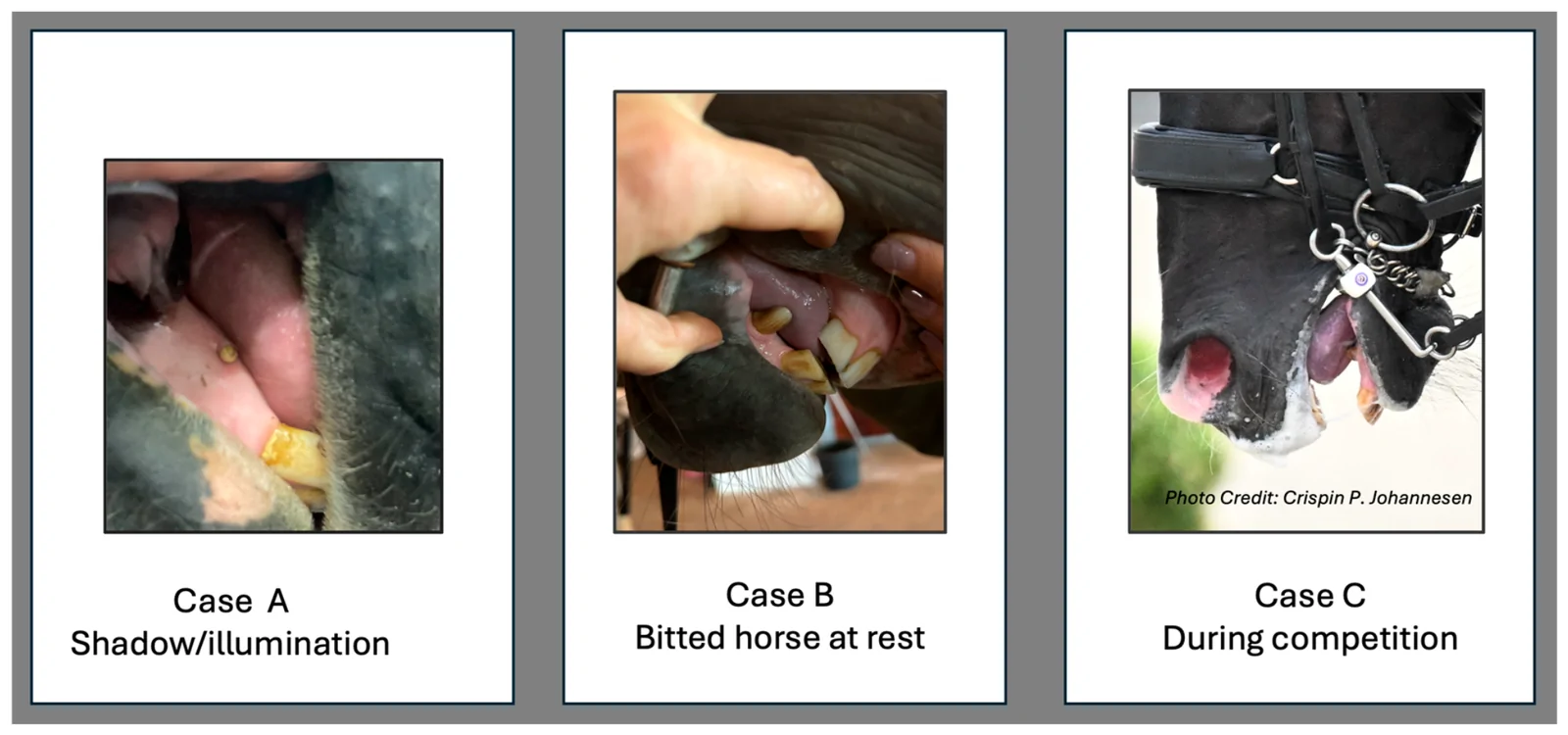
Photos of cases A–C.

Three additional cases of tongue discolouration in horses were included. Case A was a veterinary-validated photograph from a clinically normal horse in which parts of the gingival and lingual mucosae were illuminated and partly shadowed, allowing assessment of shadow artefacts. The veterinarian confirmed that the illuminated areas corresponded to the mucosal colours observed in vivo, whereas the shadowed areas did not. See Figure 2.

Case B was an owner-reported case, involving an eight-year-old Danish Warmblood gelding that developed apparent tongue discolouration while standing at rest with a bit in place for approximately 10–15 min before riding, with the head approximately at heart level. See Figure 2.

Case C comprised a veterinary-validated photograph of a healthy international-level dressage horse displaying an open mouth and visible tongue discolouration during competition (photo credit: Crispin P. Johannesen). A veterinarian positioned beside the photographer confirmed that the photographic mucosal colours corresponded to those observed directly in vivo, thereby providing the clinical validation principle used for the study photographs. See Figure 2.

Original photographs, relevant background information, informed consent and permission for scientific publication were obtained for all three cases.

### 2.6. Colour Sampling and Classification

**Figure 3:**
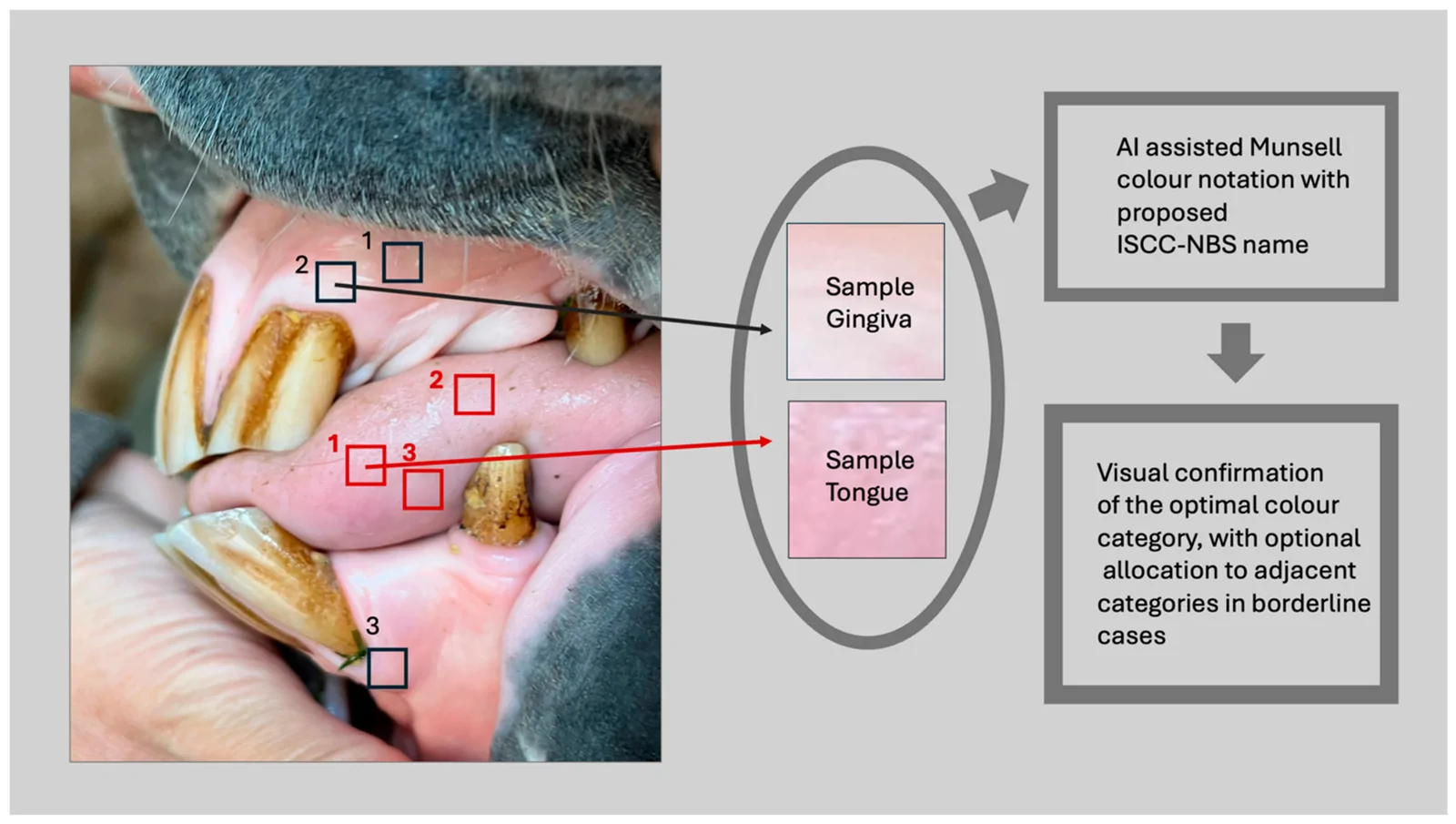
Colour samples for combined AI-assisted and visual analysis were obtained from the pre-defined anatomical regions (1, 2 or 3) in both the gingival and lingual mucosa.

At the analytical stage, one colour sample was extracted from each of the gingival and lingual mucosae from one of three predefined anatomical regions in clinically normal and sedated horses (Figure 3). The anatomical region was selected for optimal illumination and absence of shadow, reflection or other artefacts. In horses with systemic pathology, if data quality allowed it, samples were extracted from all three regions of both mucosae because patchy mucosal discolouration was observed (Figure 3), yielding up to six samples per horse. Such mottling is associated with heterogeneous microcirculatory perfusion [28,59].

Samples from the three study groups and Case C were excluded if motion blur, shadow, glare, incomplete tissue visibility or other artefacts were identified. Artefactual areas were intentionally retained in Cases A and B in line with their illustrative purposes.

Colour samples were organised according to the Uldahl Standard colour groups by visual assessment, followed by a supportive analysis using a large language model (ChatGPT, GPT-5.2, OpenAI, San Francisco, CA, USA) with the prompt “Please provide the Munsell code and ISCC-NBS colour name for the colour in the attached sample”. Munsell hue, value and chroma, as well as the corresponding ISCC-NBS colour name, were recorded. Following this procedure, each sample was assigned a formal qualitative descriptor for saturation, primary colour and, where applicable, secondary colour according to the Uldahl Standard [5]. After the initial classification, data were organised by ISCC-NBS colour name, study group and anatomical site.

A second round of visual quality control was then performed within each of the 42 colour-saturation subgroups identified during the initial classification. Samples considered visually inconsistent with the other samples in their subgroup, or borderline between classifications, underwent further assessment.

For these samples, a detailed visual comparison between relevant colour subgroups was first performed to determine the most appropriate alternative classification. AI-assisted evaluation was then repeated using the prompt “Is it possible to classify the sample under an alternative ISCC-NBS colour name [colour name]?”, with the alternative colour name based on this second-round visual assessment.

Alternative classification involved either adjustment of saturation by one category or reversal of primary and secondary colour dominance. Samples were re-assigned when the alternative visually determined classification was also accepted by the AI-assisted analysis.

Cases A–C were analysed using the same methodology in accordance with the Uldahl Standard [5].

### 2.7. Data Analysis

Descriptive analyses were performed separately for gingival and lingual samples and included clinically normal horses, sedated horses, horses with systemic pathology and the individual Cases A–C.

For each group, the number and distribution of colour-saturation combinations, predominant primary and secondary colours, saturation levels, and absent categories were summarised.

For horses with systemic pathology, multiple samples from the predefined gingival and lingual anatomical sites were included to capture the patchy and spatially heterogeneous mucosal discolouration observed in these horses.

The descriptive analysis provided an overview of colour characteristics within each category and served as a basis for subsequent comparative analyses.

Comparative statistical analyses included clinically normal and sedated horses only. Gingival and lingual samples were analysed in completely separate statistical models, with each horse contributing one observation to each anatomical region-specific analysis (gingival and lingual). Exact chi-square tests evaluated associations between physiological state and primary colour, secondary colour, and saturation separately for gingival and lingual samples (Proc. Freq, exact chi-square option, SAS 9.4 for Windows). Logistic regression was subsequently used to evaluate associations between recorded colour characteristics and physiological status and their potential predictive value (Proc Genmod, Likelihood Ratio Test, SAS 9.4 for Windows). *p*-Values below 0.05 were evaluated as significant for both chi-square tests and logistic regressions.

Primary colours were first evaluated individually, with sensitivity and specificity calculated for prediction of group status. Primary and secondary colours were then evaluated together to determine whether secondary colour improved predictive value performance. Corresponding analyses were performed for saturation classes.

## 3. Results

### 3.1. Inclusion and Exclusion of Data

In total, 63 clinically normal horses (63 gingival and 63 lingual samples), 53 sedated horses (53 gingival and 53 lingual samples) and 5 horses with systemic pathology (15 gingival and lingual samples) were included.

Due to poor image quality, 6 gingival samples (9.5%) and 11 lingual samples (17.5%) were excluded from clinically normal horses; one gingival sample (1.9%) and three lingual samples (5.7%) from sedated horses; and two gingival samples (13.3%) and seven lingual samples (46.7%) from horses with systemic pathology.

### 3.2. Descriptive Analysis of the Three Study Groups

**Figure 4:**
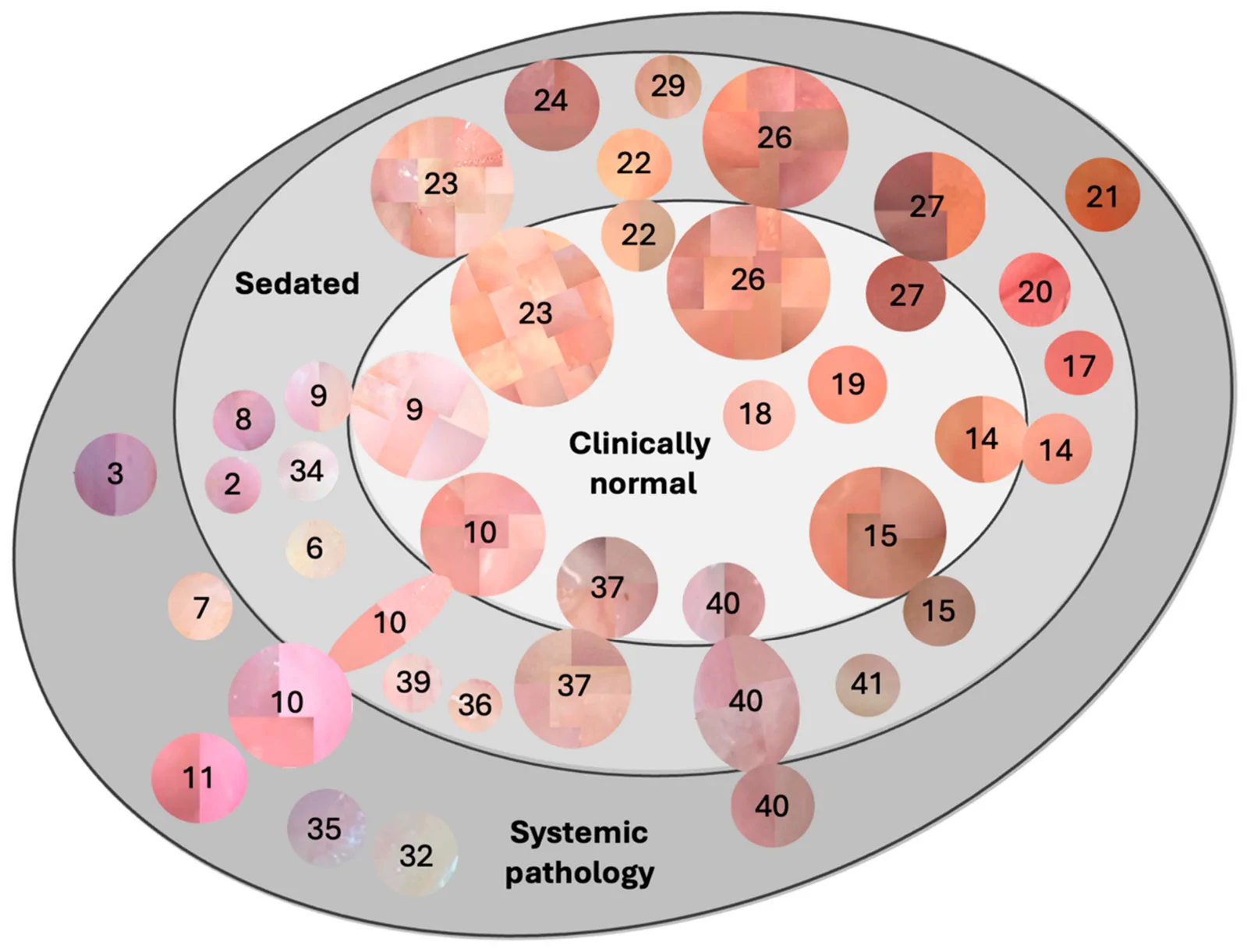
Distribution of colour classifications and saturation levels of gingival mucosae across clinically normal horses, sedated horses, and horses with systemic pathology. The number in each circle corresponds to the colour number and name in Table 1.

**Figure 5:**
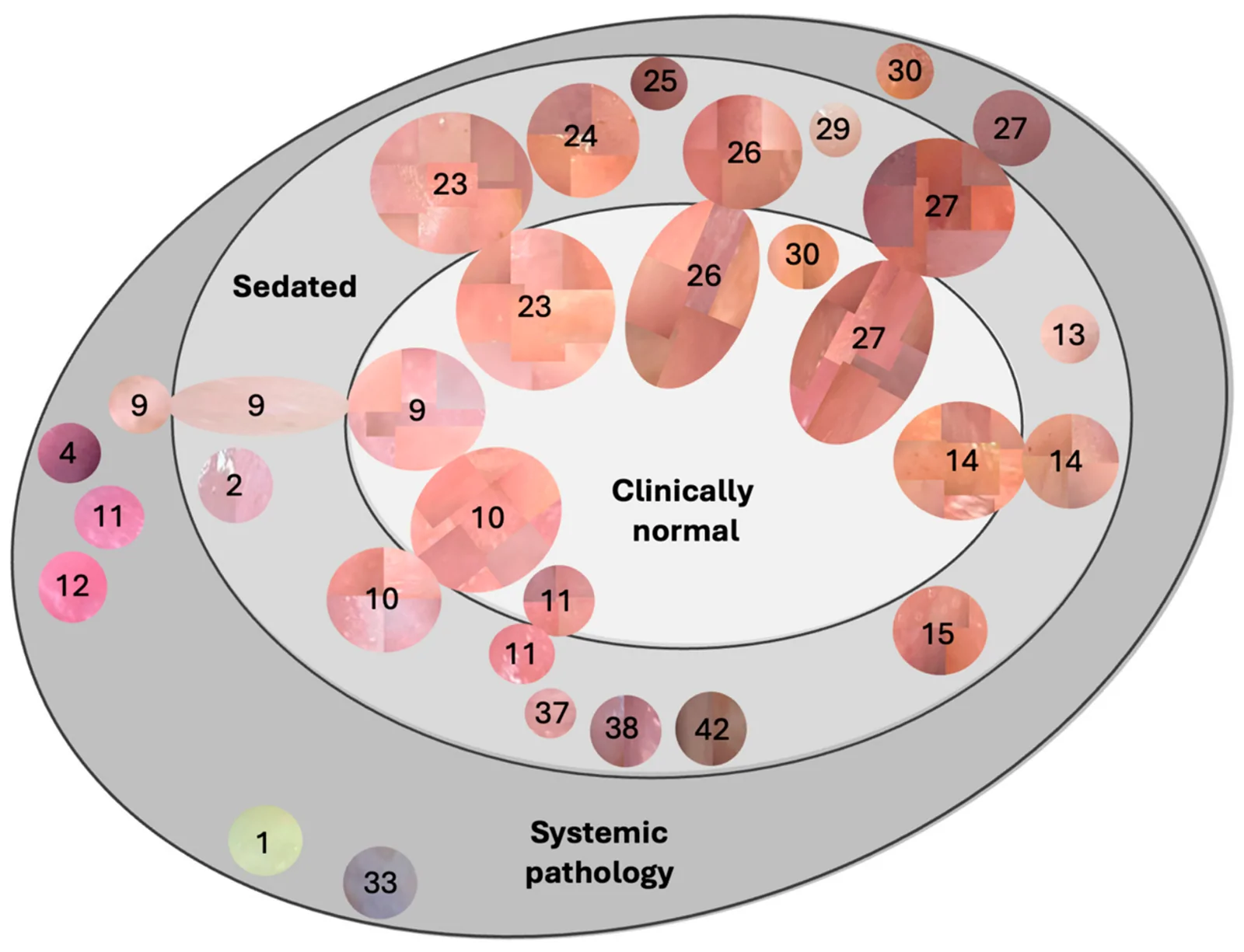
Distribution of colour classifications and saturation levels of lingual mucosae across clinically normal horses, sedated horses, and horses with systemic pathology. The number in each circle corresponds to the colour number and name in Table 1.

Overall, 42 combinations of oral equine mucosal colour and saturation were identified and presented in Table 1; they represented 42 of the 648 theoretical possible combinations defined by the Uldahl Standard (Table 1; Figure 4 and Figure 5).

#### 3.2.1. Gingival Mucosa

**Figure 6:**
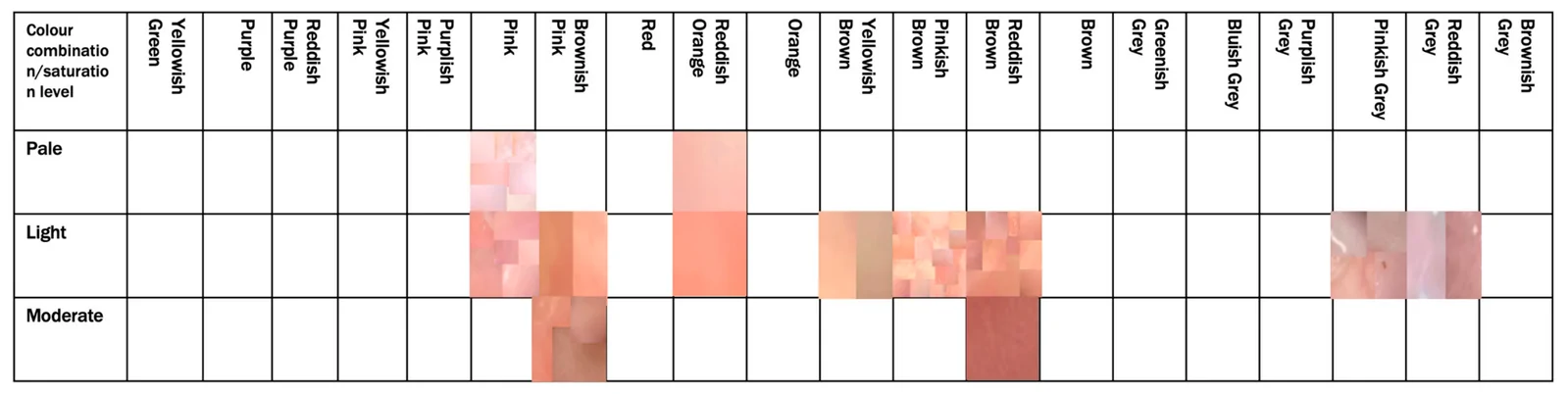
Representation of gingival mucosal colour spectrum in clinically normal horses. Strong, vivid, deep, dark or very dark levels of saturation were not observed.

Clinically normal horses (n = 57) exhibited 12 colour-saturation results. The most prevalent were light pinkish brown (29.8%; 17/57), light reddish brown (19.3%; 11/57), pale pink (12.3%; 7/57) and light pink (10.5%; 6/57). Across primary and secondary colours, pink (68.4%; 39/57) and brown (63.2%; 36/57) predominated. Light saturation was most frequent (77.5%; 45/57), followed by pale (14.0%; 8/57) and moderate (6.9%; 4/57). Green, blue and purple colours were not observed, nor were strong, vivid, deep, dark or very dark levels of saturation. See Figure 6 and Table 2.

**Figure 7:**
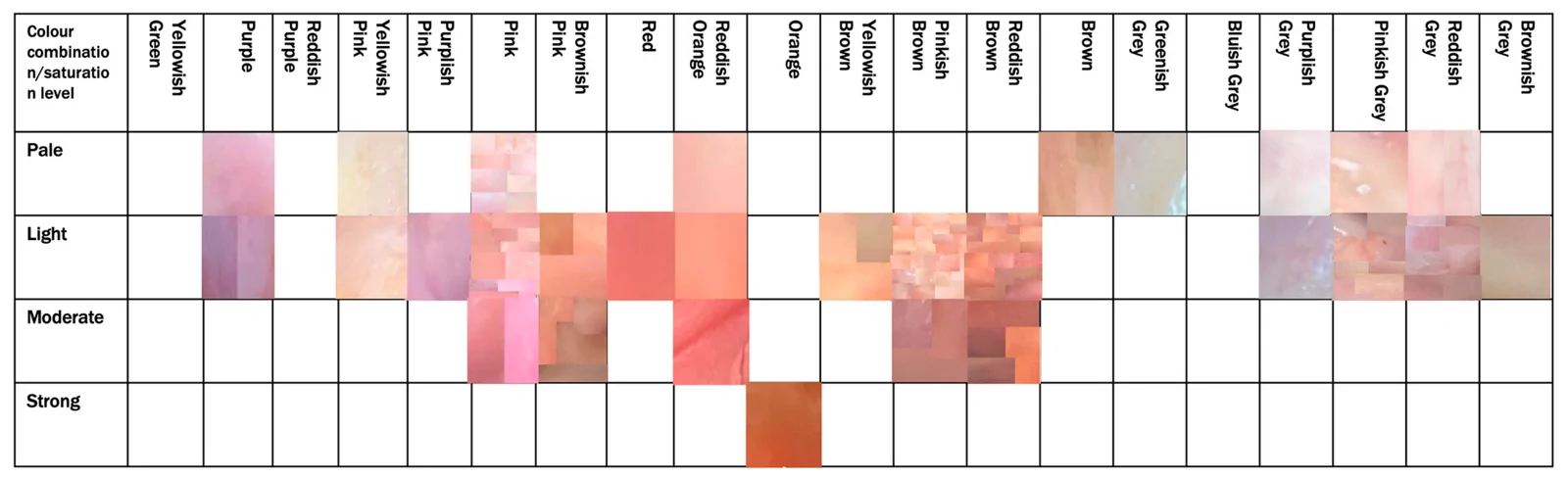
Overall representation of the gingival mucosal colour spectrum across the three groups: clinically normal horses, sedated horses and horses with systemic pathology. Vivid, deep, dark or very dark levels of saturation were not observed.

Sedated horses (n = 52) exhibited greater diversity, with 21 colour-saturation combinations. Light pinkish brown (19.2%; 10/52) and light reddish brown (15.4%; 8/52) were the most prevalent. Brown (57.7%; 30/52), pink (52.0%; 27/52) and red (36.5%; 19/52) predominated across primary and secondary colours. Light saturation was most frequent (65.4%; 34/52), followed by pale (19.2%; 10/52) and moderate (15.4%; 8/5). Purple and red occurred as primary colours, whereas green and blue were absent. Nor were strong, vivid, deep, dark or very dark levels of saturation observed. See Figure 7 and Table 2.

Horses with systemic pathology contributed 13 gingival samples comprising eight colour-saturation combinations. Pink was the predominant colour (38.5%; 5/13), but the overall distribution was heterogeneous and included purple, orange, grey and green-associated combinations. Blue and brown were not observed. Light saturation predominated (69.2%), with moderate (15.4%), pale (7.7%) and strong (7.7%) also represented. See Figure 7 and Table 2.

#### 3.2.2. Lingual Mucosa

Clinically normal horses (n = 52) exhibited eight colour saturation combinations, restricted primarily to pink and brown hues. The most prevalent were light pink and light reddish brown (both 17.3%; 9/52), followed by light brownish pink and light pinkish brown (both 15.4%; 8/52).

**Figure 8:**
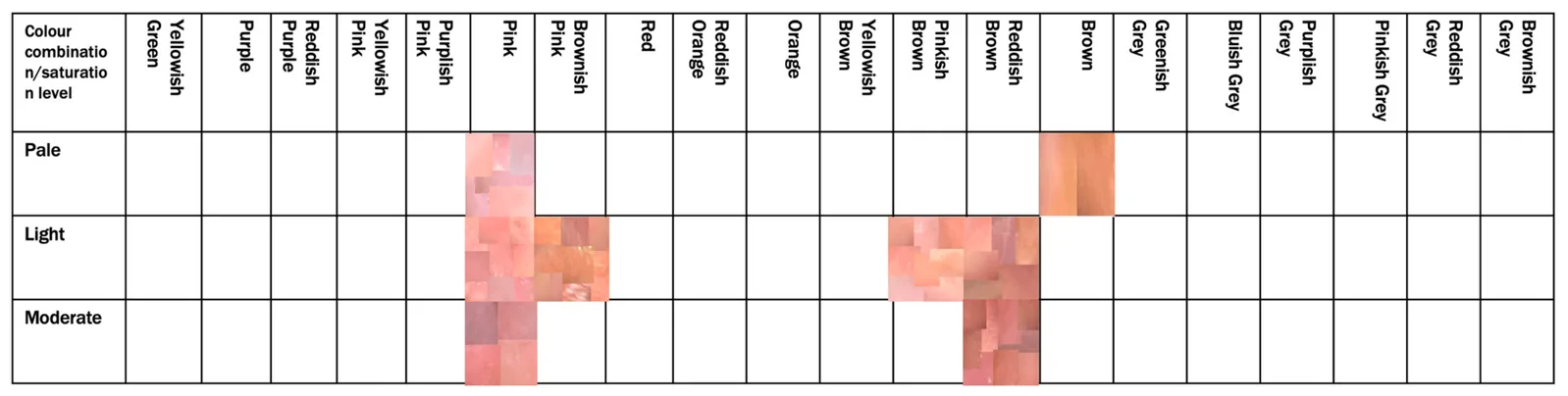
Representation of lingual mucosal colour spectrum in clinically normal horses. Strong, vivid, deep, dark or very dark levels of saturation were not observed.

Brown (63.5%; 33/52) and pink (51.9%; 27/52) predominated, and light saturation was most frequent (63.5%; 33/52), followed by moderate (25.0%; 13/52) and pale (11.5%; 6/52). Yellow, green, blue, purple, orange and grey were absent. Nor were strong, vivid, deep, dark or very dark levels of saturation observed. See Figure 8 and Table 2.

**Figure 9:**
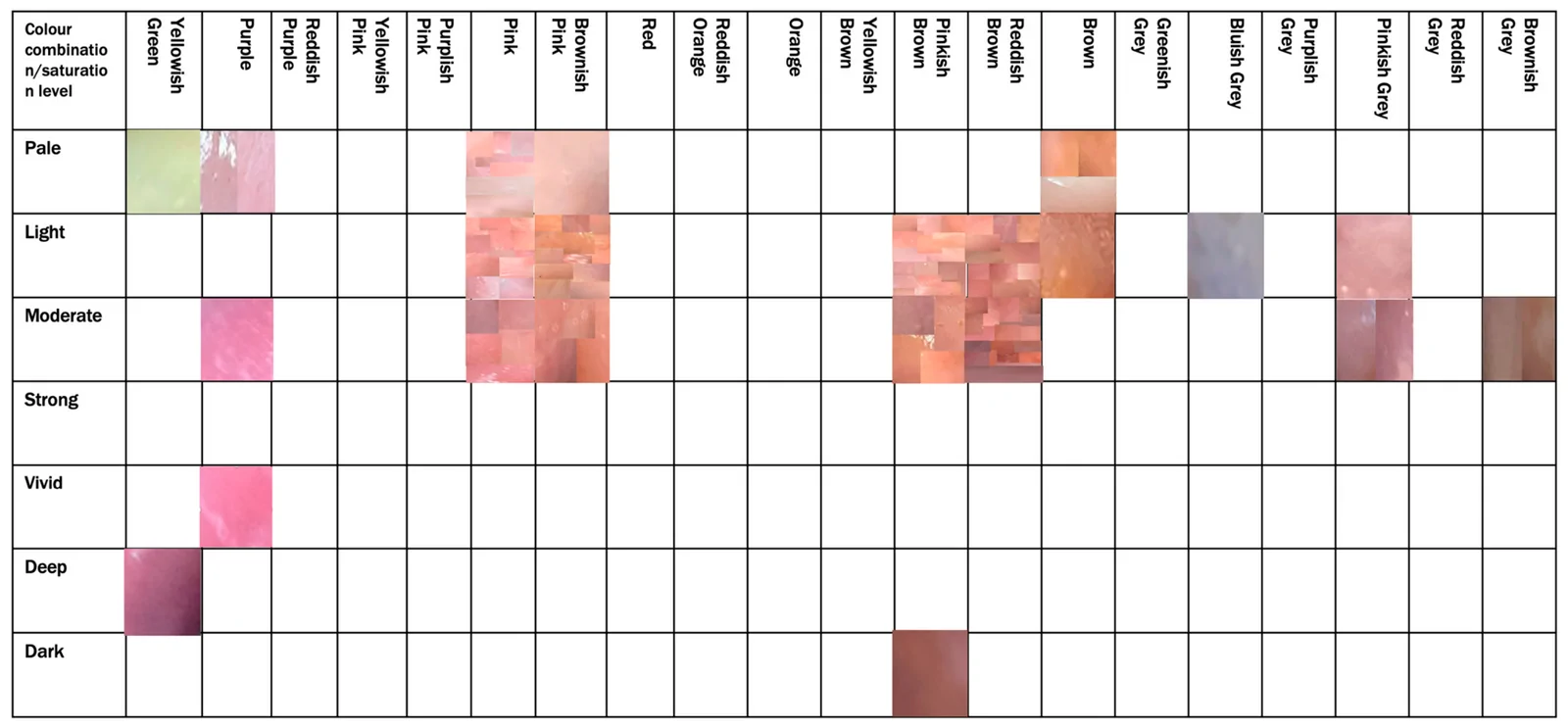
Overall representation of lingual mucosal colour spectrum across the three groups: clinically normal horses, sedated horses and horses with systemic pathology. Strong or very dark levels of saturation were not observed.

Sedated horses (n = 50) exhibited 16 combinations. Light pinkish brown (18.0%; 9/5), moderate reddish brown (16.0%; 8/50) and light reddish brown (12.0%; 6/50) were the most prevalent. Brown (78.0%; 39/50) and pink (62.0%; 31/50) predominated. Light saturation occurred in 48.0% (24/50), moderate in 40.0% (20/50), pale in 10.0% (5/50), and dark in 2.0%. Grey and purple occurred as primary colours. Yellow, green, blue and orange were not observed. Nor were strong, vivid, deep or very dark. See Figure 9 and Table 2.

The eight lingual samples from horses with systemic pathology represented eight different colour-saturation combinations, demonstrating marked heterogeneity. Overall, pink was the most common (37.5%; 3/8), while green, purple and grey also occurred as primary colours. Pale, light and moderate saturation each accounted for 25% (2/8), with vivid and deep saturation each occurring in one sample (12.5%). Yellow, blue and red occurred exclusively as secondary colours. Orange was not recorded. See Figure 9 and Table 2.

### 3.3. Comparison of Clinically Normal Horses and Sedated Horses

#### 3.3.1. Gingival Colour

The distribution of primary gingival colours differed between clinically normal and sedated horses (exact chi-square, *p* = 0.05), whereas secondary colour did not (*p* = 0.28).

In logistic regression, grey was the only primary colour significantly associated with group status (*p* = 0.05), occurring more frequently in sedated horses. Grey had a sensitivity of 27% and specificity of 89% for identifying sedated horses. No other primary colour was significantly associated with group status (Table 3).

Pink showed low sensitivity (15%) and limited specificity (68%), while brown had similar prevalence in sedated (52%) and clinically normal horses (54%; *p* = 0.85). Orange was uncommon and similarly distributed between groups (2% vs. 4%; *p* = 1.0). Purple and red occurred exclusively in sedated horses, but were uncommon, and their associations were not significant (*p* = 0.47). See Table 2.

#### 3.3.2. Lingual Colour

The distribution between primary lingual colours differed between clinically normal and sedated horses (exact chi-square, *p* = 0.004), whereas secondary colour did not (*p* = 0.60).

In logistic regression, grey (*p* = 0.03) and pink (*p* = 0.02) were significantly associated with group status. Grey occurred in 10% of sedated horses and was absent in clinically normal horses, corresponding to 10% sensitivity and 100% specificity for identifying sedated horses in this dataset. Pink had low sensitivity (28%) and poor specificity (48%), being present in 52% of clinically normal horses. Grey remained significantly associated with physiological state when primary and secondary colours were analysed together (*p* = 0.03), whereas the association with pink did not (*p* = 0.68). No other primary colour was significantly associated with group status. See Table 3.

Brown was common in both groups (58% sedated vs. 48% clinically normal; *p* = 0.33). Purple occurred exclusively in sedated horses (4%) but was uncommon and not significantly associated with group status (*p* = 0.24). See Table 2.

#### 3.3.3. Saturation

Saturation did not differ significantly between clinically normal and sedated horses for either gingival (*p* = 0.25) or lingual mucosae (*p* = 0.10). Moderate saturation was numerically more frequent in sedated horses for both gingival (66.7% of moderate samples) and lingual mucosa (60.6%).

Primary colour was significantly associated with saturation in both gingival (*p* = 0.002) and lingual samples (*p* = 0.02). However, separate analyses of pink and brown showed no significant colour-saturation associations for gingival pink (*p* = 1.0), gingival brown (*p* = 0.32), lingual pink (*p* = 0.44) or lingual brown (*p* = 0.47).

### 3.4. Colour and Saturation Analysis of Three Individual Cases

The three individual cases were analysed to illustrate the practical application of the colour classification framework across different specifically defined scenarios. In Case A, samples were obtained from both illuminated and corresponding shadowed areas of the gingival and lingual mucosae. The illuminated gingival and lingual samples were both classified as light yellowish pink, whereas the corresponding shadowed samples were moderate brown and dark reddish brown, respectively.

In Case B, both samples were obtained from shadowed areas. The gingival mucosa was classified as moderate brownish pink, and the lingual mucosa as dark brownish pink.

**Figure 10:**
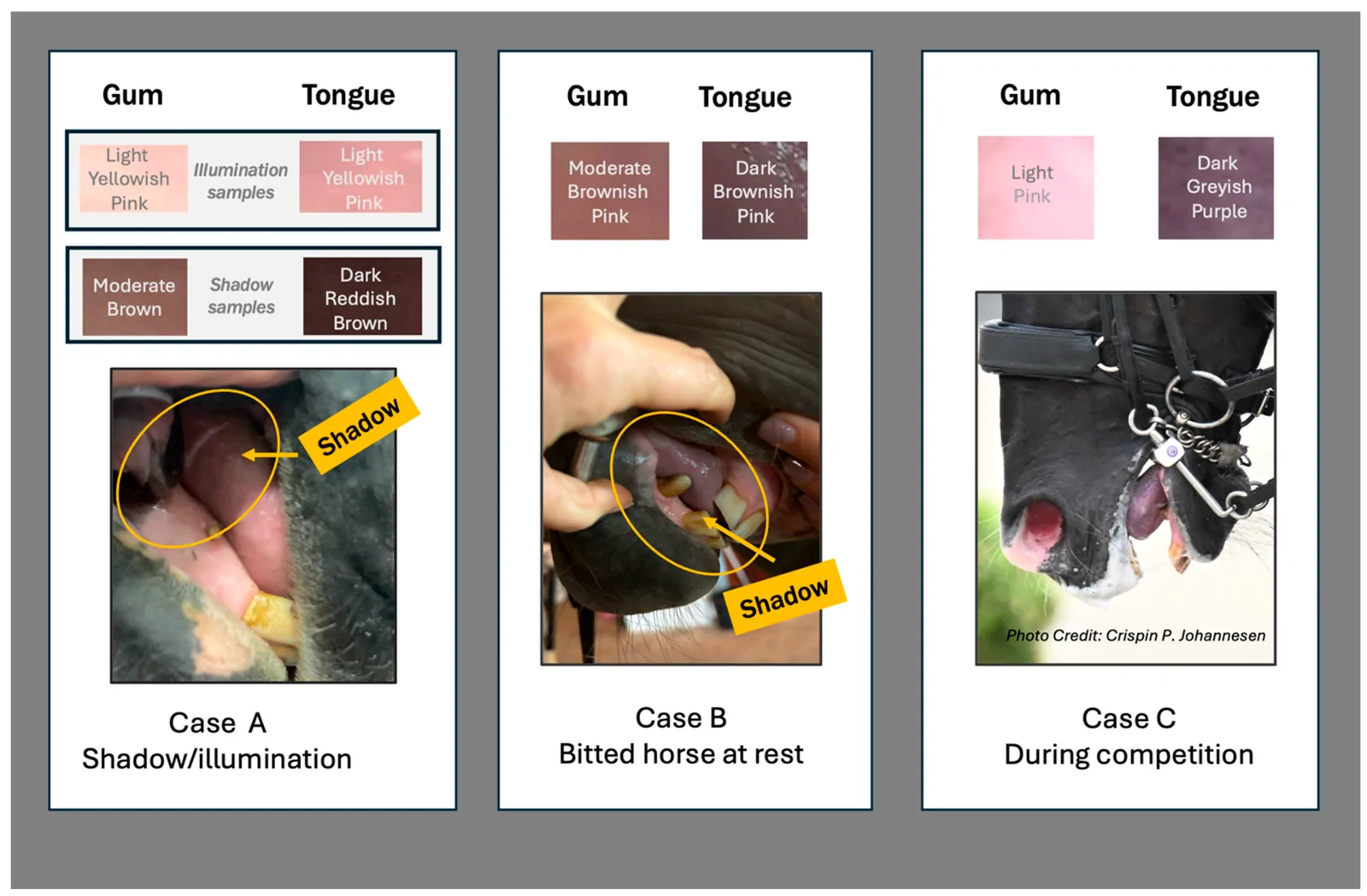
Photographs of horses included in Cases A–C with corresponding colour samples and ISCC-NBS colour names.

In Case C, both samples were obtained from illuminated areas. The gingival mucosa was classified as light pink, and the lingual mucosa as dark greyish purple (Figure 10; Table 1).

## 4. Discussion

This study provides an initial proof of concept demonstrating that the Uldahl Standard can be applied to equine mucosal colour assessment. Rather than seeking to establish an exhaustive diagnostic colour classification, the study demonstrates the feasibility of applying a standardised framework to describe mucosal colour and saturation with greater precision than has previously been possible.

This was demonstrated at two levels. First, the framework was used to characterise the range of colours observed across different physiological and pathological groups. Overall, 42 unique colour-saturation combinations were identified, illustrating considerable chromatic diversity and indicating that not all observed variations are likely to be diagnostically informative.

Second, the study demonstrates how statistical analysis can be used to distinguish potentially informative from non-informative colour characteristics in relation to physiological state. This is exemplified by applying the framework to non-sedated and sedated clinically normal horses using comparative and logistic regression analyses to further identify predictors associated with physiological alteration.

Distinguishing such informative characteristics from the broader spectrum of naturally occurring colour variation represents an important step towards development and validation of robust diagnostic criteria.

As a third component of the study, the individual cases illustrated application of the framework to photographic material and welfare-related observations. Together, these findings provide methodological guidance for future studies investigating associations between specific colour patterns and well-defined physiological and pathological conditions.

An important feature of the present approach was that the veterinary visual assessment remained the primary method applied to both the clinical validation and analytical stages. Direct in vivo assessment by veterinarians served as the reference for determining whether the photographic representation adequately reflected the observed mucosal colour, rather than treating the photograph as an objective colour measurement—a strategy that is consistent with validation protocols reported in other fields [51,52,53,54,55].

At the subsequent analytical stage, visual assessment and allocation of samples to the principal colour categories were likewise undertaken through visual assessment performed by a veterinarian. Generative AI was used as a secondary supportive tool to facilitate more detailed interpretation of colour characteristics and their relation to Munsell notation and ISCC-NBS terminology.

This approach is consistent with emerging human-AI collaborative models in other scientific and clinical fields, in which generative AI supplements expert assessments merely as a tool, while the human expert remains responsible for the primary assessment and final interpretation [47,48,49,50].

### 4.1. Chromatic Variation and Physiological State

Chromatic diversity increased with physiological compromise. Both gingival and lingual mucosae showed greater colour variation in sedated horses than in non-sedated clinically normal horses. The few horses with systemic pathology showed the greatest heterogeneity. Gingival mucosae were generally more chromatically diverse than lingual mucosae, particularly under sedation.

Primary colour differed significantly between clinically normal and sedated horses, whereas secondary colour and saturation did not, suggesting that within the present dataset, primary colour appeared to be the more informative level of the three characteristics for identifying physiological alteration. However, contributions from secondary colour and saturation cannot be excluded.

### 4.2. Diagnostic Relevance of Colour and Saturation

Grey showed the greatest diagnostic relevance. It was significantly associated with sedation status and was also observed in the few horses with systemic pathology. Although sensitivity was low, specificity was high, suggesting that grey functions better as a confirmatory indicator than as a screening marker. Grey mucosal colour has not been studied as an isolated diagnostic finding in horses, but has been linked to impaired tissue perfusion and oxygen delivery [25,59].

Pink and brown were common across groups and consequently showed limited discriminatory value.

Purple and blue occurred only in sedated horses and in the few horses with systemic pathology, consistent with previous associations with impaired oxygenation, venous congestion, and circulatory disturbance. However, their low frequency prevented robust statistical evaluation.

Yellow, red, orange, and green were also uncommon and showed limited diagnostic value in the present dataset, although their occurrence was broadly consistent with processes described in the literature, including transient yellow discolouration in otherwise healthy fasting horses [61].

Saturation was not significantly associated with physiological state but was associated with primary colour. Light saturation predominated in both clinically normal and sedated horses, while moderate saturation occurred numerically more frequently in sedated horses in both gingival and lingual samples. Saturation may therefore provide complementary information, although its diagnostic value appears secondary to primary colour. This interpretation is consistent with previous clinical literature in which increasingly intense or darker mucosal colouration has been associated with greater severity of physiological compromise or pathology, as exemplified by Hildebran et al. [26].

Pain was not assessed in the present study. However, a biologically plausible relationship between circulatory disturbance, cutaneous or mucosal colour change, tissue integrity and pain has been described [5].

Evidence from other mammalian species indicates that restricted venous and lymphatic outflow may cause congestion, oedema and accumulation of deoxygenated blood, with colour progressing from red towards purple or blue as circulatory compromise develops [15,59]. Restricted blood flow contributes to pain through tissue distension, ischaemic activation of nociceptors, and nerve compression or injury [62,63,64,65], with erythema and purple to dusky-purple discolouration associated with increasing pain severity in some clinical contexts [15,65]. With advancing arterial compromise, colour may progress towards grey mottling and eventually black necrotic tissue [66,67]. In horses, tourniquet-induced ischaemia has been used as a controlled nociceptive stimulus [68].

Collectively, these findings provide a rationale for investigating potential relationships among mucosal colour change, circulatory compromise, tissue integrity and pain in horses.

### 4.3. Standardised Terminology and Clinical and Welfare Implications

The colour categories identified in the present study corresponded closely to those described in the veterinary literature and were fully represented within the Uldahl Standard. Formal qualitative descriptors used in the literature generally aligned with its saturation classes. Standardisation of colour and saturation terminology may, therefore, improve clinical communications and facilitate comparisons between studies.

The framework is intended to accommodate different validation levels according to purpose: veterinary clinical validation may support routine clinical application, whereas calibrated photographic or instrumental approaches may be appropriate when quantitative measurement is required.

The individual cases further demonstrated the practical value of structured colour assessment. Cases A and B illustrated the substantial influence of shadow on photographic interpretation. In Case C, the marked contrast between the normally coloured gingival mucosa and the dark greyish-purple lingual mucosa suggested a localised disturbance affecting the tongue rather than a generalised alteration in systemic mucosal colour. Given the absence of reported clinical evidence of systemic disease, the lingual discolouration was considered consistent with local venous congestion, which may produce dark red to purple discolouration through impaired venous outflow and accumulation of poorly oxygenated blood [4,5,15,22,59,65]. Venous congestion and insufficient blood supply have been associated with pain [5,15,68], while progression from pink to purple or blue implicates increasing physiological compromise [15,26,59].

Together, the individual cases demonstrate how structured assessment may support interpretation and validation of photographic material, and thereby may contribute to both clinical and welfare evaluation. They also illustrate why the validation level and the relationship between photographic representation and direct in vivo observation should be reported explicitly.

### 4.4. Additional Factors Potentially Influencing Mucosal Colour

Interpretation of differences in mucosal colour between physiological states requires consideration of factors that may influence mucosal appearance independently of disease or physiological compromise. Exercise represents one such factor, as altered mucosal appearance has been reported following exercise in polo and endurance horses. However, with the exception of Case C, all horses in the present study were assessed under resting conditions.

Age and sex may also contribute to variation in mucosal appearance, although current evidence is limited. In addition, the potential influence of medications administered to the five horses with severe systemic pathology was not accounted for.

These factors were not included in the present analyses, because this proof-of-concept study was designed to provide an initial characterisation of mucosal colour across the three broad clinical categories: clinically normal, sedated and horses with systemic pathology. These broad groupings were used to explore whether clinically relevant differences in mucosal colour could be identified before introducing additional demographic, physiological and treatment-related variables. Future studies can build on these findings by refining the clinical categories.

### 4.5. Methodological Considerations and Future Directions

The photographic material was obtained under variable clinical and environmental conditions and was not standardised with respect to smartphone model, controlled illumination, ambient illuminance (lux), flash settings or grey card calibration.

Importantly, the photographs were not treated as objective instrumental measurements of mucosal colour. Instead, this proof-of-concept study applied conventional veterinary clinical validation: veterinarians assessed mucosal colour directly in vivo, and only photographs considered to adequately represent the observed colour were eligible for analysis. This reflects the routine clinical context in which mucosal colour is assessed visually without instrumental measurement of illumination. Nevertheless, both colour perception and photographic representation may be influenced by lighting, shadow, equipment, and individual perception, which constitute limitations of the present approach. Cases A and B specifically illustrated the substantial effect of shadow on photographic colour representation.

Veterinary visual assessment remained the primary method at the analytical classification stage, with generative AI used as a secondary supportive tool for more detailed colour characterisation. Potential limitations and biases inherent to generative AI should therefore be considered in relation to its use here in a supplementary role as opposed to a questionable use as an autonomous diagnostic or colourimetric method.

Future studies incorporating calibrated photography or deterministic digital colourimetry, as outlined in the Uldahl Standard [5] could provide an additional and more objective level of validation. Such technological refinement should, however, be considered alongside the accessibility, clinical applicability, and established role of veterinary visual assessment in routine practice and horse welfare evaluation.

Further validation should include larger populations representing well-defined physiological and pathological conditions, with particular attention to grey, blue and purple and their relationships with circulatory impairment and pain. Demographic and individual factors should also be evaluated as potential influences on mucosal colour.

Integrating colour assessment with objective physiological measurements and, where appropriate, calibrated photographic or instrumental colour measurement could help establish evidence-based reference ranges and determine the diagnostic performance of specific colour-saturation patterns across different levels of validation.

## 5. Conclusions

This study provides initial proof of concept for applying the Uldahl Standard to equine oral mucosal colour assessment. The framework enabled a systematic description of colour and saturation across clinically normal and sedated horses, as well as horses with systemic cardiovascular compromise, identifying 42 unique colour-saturation combinations.

Chromatic diversity increased with physiological compromise, and primary colour showed greater discriminatory potential than secondary colour or saturation. Grey was associated with physiological compromise and showed high specificity, while purple and blue occurred only in compromised horses in the present dataset and were also represented by the dark greyish-purple tongue in case C. Saturation alone was not significantly associated with physiological state but may provide complementary information.

Rather than establishing an exhaustive diagnostic classification, these findings provide a methodological foundation for distinguishing potentially informative from non-informative colour characteristics. The Uldahl Standard offers standardised terminology and a structured approach for comparing clinical observations, photographic material and future research. Larger studies of well-defined physiological and pathological conditions are required to further determine the diagnostic relevance of specific colour-saturation patterns.

## Figures and Tables

**Table 1:** Distribution of ISCC-NBS colour categories and corresponding Munsell notations in gingival and lingual mucosa across the examined horse groups and cases.

Colour No.	ISCC-NBS Name	Gingival Mucosa		Lingual Mucosa	
		Munsell Notation	No. of Samples	Munsell Notation	No. of Samples
1	Pale Yellowish Green	-	-	5Y 8/4 C	1C
2	Pale Purple	5P 7/3 S	1S	5P 7/3 S 5RP 8/2 S	2S
3	Light Purple	5RP 7/4 C	2C	-	-
4	Deep Reddish Purple	-	-	5RP 5/6 C	1C
5	Dark Greyish Purple	-	-	5RP 4/2 X	1X
6	Pale Yellowish Pink	10YR 8.5/2 S	1S	-	-
7	Light Yellowish Pink	5YR 8/2 C 5YR 8/3 X	1C 1X	5YR 7/4 X	1X
8	Light Purplish Pink	7.5RP 7/4 S	1S	-	-
9	Pale Pink	5YR 8/2 N 5YR 8/4 N 5YR 8.5/2 N 5YR 9/2 N 7.5YR 8/4 N 5RP 8/2 S 5R 8/2 N 5R 8/3 S 5R 9/2 N	7N 2S	5YR 8/2 C 5YR 8/4 N 5YR 8.5/2 S 7.5YR 8.5/2 N 5R 8/2 N 5R 8/3 N 5R 8/4 N 7.5R 8/4 N	6N 1S 1C
10	Light Pink	5R 7/4 N + S + C 5R 8/3 S 5R 8/4 N + C 7.5RP 7/4 X	6N 2S 3C 1X	5R 7/4 N + S 5R 8/4 N + S 7.5R 7/4 N 7.5R 8/4 N	9N 4S
11	Moderate Pink	5R 6/6 C 5R 7/6 C	2C	5R 6/4 N 5R 6/6 N + S 5R 7/6 C	4N 1S 1C
12	Vivid Pink	-	-	5R 7/8 C	1C
13	Pale Brownish Pink	-	-	5YR 7/3 S	1S
14	Light Brownish Pink	5YR 7/4 S 7.5YR 6/4 N	2N 1S	5YR 6/4 N + S 5YR 7/4 N + S 7YR 6/4 S 7.5YR 7/4 N + S	8N 4S
15	Moderate Brownish Pink	5YR 4/4 X 5YR 5/3 S 5YR 6/4 N 7.5YR 5/4 N	3N 1S 1X	5YR 5/4 S 5YR 6/4 S	3S
16	Dark Brownish Pink	-	-	5YR 3/4 X	1X
17	Light Red	5R 6/4 S	1S	-	-
18	Pale Reddish Orange	5R 8/4 N	1N	-	-
19	Light Reddish Orange	7.5R 7/6 N	1N	-	-
20	Moderate Reddish Orange	5R 5/6 S	1S	-	-
21	Strong Orange	5YR 6/8 C	1C	-	-
22	Light Yellowish Brown	5YR 7/4 N 5YR 7/6 S 10YR 8/4 N	2N 1S	-	-
23	Light Pinkish Brown	5YR 6/4 S 5YR 7/4 N + S 5YR 8/3 S 5YR 8/4 N 5YR 8.5/3 N + S 7.5YR 7/4 N 7.5YR 7.5/3 S 7.5YR 8/3 N + S 7.5YR 8/4 N 7.5YR 8.5/4 N	17N 10S	5YR 6/4 S 5YR 7/4 N + S 5YR 8/4 S 5YR 8.5/3 N 7.5YR 7/4 N + S 7.5YR 8/4 N 7.5YR 8.5/3 N	8N 9S
24	Moderate Pinkish Brown	5YR 5/3 S 5YR 5/4 S	3S	5YR 5/3 S 7.5YR 6/4 S 7.5YR 7/4 S	4S
25	Dark Pinkish Brown			5YR 4/3 S	1S
26	Light Reddish Brown	5 YR 6/4 N + S 5YR 6/6 S 5YR 7/4 N + S 5YR 8/4 N 5R 6/6 N + S 5R 7/4 N 7.5R 7/4 N 7.5R 7/6 N	11N 8S	5YR 6/4 S + N 5YR 7/4 S + N 5R 6/6 N 5R 7/4 N 7.5R 7/4 N	6N 6S
27	Moderate Reddish Brown	5YR 5/4 S 5YR 6/6 S 5R 5/4 S 5R 5/6 N	1N 3S	5YR 4/4 S 5YR 5/4 N + S 5R 5/4 N + S + C 5R 5/6 N 5R 6/4 N 5R 6/6 N 7.5R 6/4 N 7.5R 6/6 S	9N 8S 1C
28	Dark Reddish Brown	-	-	5YR 3/3 X	1X Shadow
29	Pale Brown	5YR 7/3 S	2S	5YR 7/3 S	1S
30	Light Brown	-	-	5YR 6/6 C 7.5 YR 6/4 N 7.5 YR 7/4 N	2N 1C
31	Moderate Brown	7.5YR 4/3 X	1X Shadow	-	-
32	Pale Greenish Grey	5BG 9/1 C	1C	-	-
33	Light Bluish Grey	-	-	5PB 7/2 C	1C
34	Pale Purplish Grey	5RP 8/2 S	1S	-	-
35	Light Purplish Grey	5P 8/2 C	1C	-	-
36	Pale Pinkish Grey	5YR 8/2 S	1S	-	-
37	Light Pinkish Grey	2.5YR 7/3 N 5YR 6/2 N + S 5YR 6/3 S 5YR 6/4 S 5YR 7/2 N 5YR 7/3 S 5YR 8/2 S	4N 5S	5YR 7/4 S	1S
38	Moderate Pinkish Grey	-	-	5YR 5/2 S	2S
39	Pale Reddish Grey	5R 8/2 S	2S	-	-
40	Light Reddish Grey	5YR 5/3 S 5YR 6/2 N + S 5R 6/4 N + C 5R 7/2 C	2N 4S 2C	-	-
41	Light Brownish Grey	5YR 7/2	1S	-	-
42	Moderate Brownish Grey			5YR 5/2 S	2S
	Total number of samples per group		57N 52S 13C 4X		52N 50S 8C 4X

N (clinically normal horses), S (sedated horses), C (horses with systemic pathology) and X (case-specific observations) denote the group identifiers used in the Munsell notation entries; - indicates that no entry was reported.

**Table 2:** Distribution of primary and secondary gingival and lingual mucosal colours according to clinical status.

Clinical Group	Colour Dominance	Anatomical Region	Yellow	Green	Blue	Purple	Pink	Red	Orange	Brown	Grey
Non-sedated	Primary	Gingiva	-	-	-	-	X	-	X	X	X
	Primary	Tongue	-	-	-	-	X	-	-	X	-
	Secondary	Gingiva	X	-	-	-	X	X	-	X	-
	Secondary	Tongue	-	-	-	-	X	X	-	X	-
Sedated	Primary	Gingiva	-	-	-	X	X	X	X	X	X
	Primary	Tongue	-	-	-	X	X	-	-	X	X
	Secondary	Gingiva	X	-	-	X	X	X	-	X	-
	Secondary	Tongue	-	-	-	-	X	X	-	X	-
Systemic Pathology	Primary	Gingiva	-	-	-	X	X	-	X	-	X
	Primary	Tongue	X	X	-	X	X	-	-	X	X
	Secondary	Gingiva	X	X	-	X	X	X	-	-	-
	Secondary	Tongue	-	-	X	-	X	X	-	-	-

X indicates that the colour category was observed in at least one horse within the corresponding clinical group and anatomical region; - indicates that the category was not observed.

**Table 3:** Significant differences in gingival and lingual mucosal colour between clinically normal and sedated horses.

Anatomical Region	Statistical Analysis	Colour/Variable	*p*-Value	Sensitivity (%)	Specificity (%)
Gingiva	Exact chi-square test	Primary colour distribution	0.05	-	-
Gingiva	Logistic regression	Grey (primary)	0.05	27 (15.6–41.0)	89 (78.5–96.0)
Tongue	Exact chi-square test	Primary colour distribution	0.004	-	-
Tongue	Logistic regression	Grey (primary)	0.03	10 (3.3–22.8)	100 (93.1–100)
Tongue	Logistic regression,	Grey (primary and secondary combined)	0.03	-	-
Tongue	Logistic regression	Pink (primary)	0.02	28 (16.2–42.5)	48 (34.0–62.4)

*p*-values ≤ 0.05 were considered statistically significant. Sensitivity and specificity are presented as percentages with 95% confidence intervals in parentheses; - indicates that sensitivity and specificity were not reported.

## Data Availability

All necessary data required for interpreting the outcomes of this work have been included in this paper.
